# CED-6/GULP and components of the clathrin-mediated endocytosis machinery act redundantly to correctly display CED-1 on the cell membrane in *Caenorhabditis elegans*

**DOI:** 10.1093/g3journal/jkae088

**Published:** 2024-05-02

**Authors:** Rikke Hindsgaul Harders, Tine H Morthorst, Line E Landgrebe, Anna D Lande, Marie Sikjær Fuglsang, Stine Bothilde Mortensen, Verónica Feteira-Montero, Helene Halkjær Jensen, Jonas Bruhn Wesseltoft, Anders Olsen

**Affiliations:** Department of Chemistry and Bioscience, Aalborg University, Fredrik Bajers Vej 7H, Aalborg, DK-9220, Denmark; Department of Molecular Biology and Genetics, Aarhus University, Gustav Wieds Vej 10C, Aarhus, DK-8000, Denmark; Department of Molecular Biology and Genetics, Aarhus University, Gustav Wieds Vej 10C, Aarhus, DK-8000, Denmark; Department of Molecular Biology and Genetics, Aarhus University, Gustav Wieds Vej 10C, Aarhus, DK-8000, Denmark; Department of Molecular Biology and Genetics, Aarhus University, Gustav Wieds Vej 10C, Aarhus, DK-8000, Denmark; Department of Chemistry and Bioscience, Aalborg University, Fredrik Bajers Vej 7H, Aalborg, DK-9220, Denmark; Department of Chemistry and Bioscience, Aalborg University, Fredrik Bajers Vej 7H, Aalborg, DK-9220, Denmark; Department of Chemistry and Bioscience, Aalborg University, Fredrik Bajers Vej 7H, Aalborg, DK-9220, Denmark; Department of Chemistry and Bioscience, Aalborg University, Fredrik Bajers Vej 7H, Aalborg, DK-9220, Denmark; Department of Chemistry and Bioscience, Aalborg University, Fredrik Bajers Vej 7H, Aalborg, DK-9220, Denmark

**Keywords:** CED-1, endocytosis, clathrin, dynein, *Caenorhabditis elegans*, CED-6/GULP, AP2, CME

## Abstract

CED-1 (cell death abnormal) is a transmembrane receptor involved in the recognition of “eat-me” signals displayed on the surface of apoptotic cells and thus central for the subsequent engulfment of the cell corpse in *Caenorhabditis elegans*. The roles of CED-1 in engulfment are well established, as are its downstream effectors. The latter include the adapter protein CED-6/GULP and the ATP-binding cassette family homolog CED-7. However, how CED-1 is maintained on the plasma membrane in the absence of engulfment is currently unknown. Here, we show that CED-6 and CED-7 have a novel role in maintaining CED-1 correctly on the plasma membrane. We propose that the underlying mechanism is via endocytosis as CED-6 and CED-7 act redundantly with clathrin and its adaptor, the Adaptor protein 2 complex, in ensuring correct CED-1 localization. In conclusion, CED-6 and CED-7 impact other cellular processes than engulfment of apoptotic cells.

## Introduction

Endocytosis is the uptake of extracellular material into the cell by invagination of the plasma membrane. The main pathway responsible for endocytosis is clathrin-mediated endocytosis (CME), where cargoes are taken up into clathrin-coated vesicles, which after shedding the clathrin-coat later fuse with endosomes for further processing of the cargoes, e.g. for degradation or recycling (Mettlen *et al*. 2018). CME serves several cellular functions, such as regulating the display of proteins on the cell surface, bringing nutrients into the cell, controlling the activation of signaling pathways, and turning over membrane components and sending them to lysosomes (Mettlen *et al*. 2018). CME consists of 5 steps: nucleation, cargo selection, coat assembly, vesicle scission, and uncoating. Clathrin does not bind directly to the plasma membrane or to the receptors being internalized. Instead, it takes advantage of various adaptor proteins to make the correct interactions. Adaptor protein 2 (AP2) is the most abundant adaptor complex in CME and binds directly to clathrin (Pearse and Robinson 1990; Blondeau *et al*. 2004). In most cases, AP2 is required for the formation of clathrin-coated pits (Motley *et al*. 2003; Huang *et al*. 2004; Boucrot *et al*. 2010). The AP2 consists of 4 different subunits, the large α and β2 subunits, and the smaller µ2 and σ2 subunits (Matsui and Kirchhausen 1990; Collins *et al*. 2002). AP2 recruits clathrin to the plasma membrane and selects cargo to be incorporated into clathrin-coated pits (Traub 2009; Boucrot *et al*. 2010). Multiple accessory adaptor proteins exist that bind to different receptors and recruit them to clathrin-coated pits, collectively these are called clathrin-associated sorting proteins (CLASPs) (Edeling *et al*. 2006; Schmid *et al*. 2006; Traub 2009). Numerous receptors are internalized through CME, with the transferrin, epidermal growth factor, and low-density lipoprotein receptors being among the best studied. Some receptors require stimulation of ligands to be endocytosed, other receptors, like the transferrin receptor, are continuously endocytosed (Hopkins *et al*. 1985). Internalized receptors can either be recycled to the plasma membrane through endosomes and the trans-Golgi network (TGN), or they can be degraded following fusion with lysosomes (Mueller *et al*. 2002; Grant and Donaldson 2009; Saftig and Klumperman 2009). Correct regulation of internalization and recycling of a receptor is important for its function and signal transduction potential.

In *Caenorhabditis elegans*, CED-1 (cell death abnormal) is a transmembrane receptor with homology to the human scavenger receptor from endothelial cells and CD91/low-density lipoprotein receptor-related protein (LRP) (Zhou *et al*. 2001; Su *et al*. 2002). CED-1 is localized in the plasma membrane of the engulfing cells and recognizes “eat-me” signals displayed on the surface of apoptotic cells (Zhou *et al*. 2001). The recognition of apoptotic cells by CED-1 is mediated by CED-7, a 12-pass membrane protein of the ATP-binding cassette transporter family (Wu and Horvitz 1998). After CED-1 binds to apoptotic cells, it transmits a signal for engulfment through its intracellular domain (Zhou *et al*. 2001; Su *et al*. 2002). The adaptor protein CED-6 is a downstream mediator of CED-1 signaling (Su *et al*. 2002). GULP is the human homolog of CED-6, and it also promotes phagocytosis in humans (Liu and Hengartner 1999; Smits *et al*. 1999; Su *et al*. 2002). CED-6 contains a phosphotyrosine-binding (PTB) domain (Liu and Hengartner 1998), which interacts directly with the NPXY domain of CED-1 (Su *et al*. 2002). Mutations in either CED-1, CED-6, or CED-7 cause engulfment defects in both somatic tissues and in the germline of *C. elegans*. How CED-1 mediates engulfment has been intensively studied and numerous proteins have been found acting downstream of CED-1 in the engulfment of apoptotic cells (Zhou *et al*. 2001; Su *et al*. 2002; Yu *et al*. 2006; Kinchen *et al*. 2008; Almendinger *et al*. 2011; Lu *et al*. 2011; Chen *et al*. 2013; Fancsalszky *et al*. 2014). CED-1 is recycled to the plasma membrane after engulfment by the retromer (Lu *et al*. 2011) complex proteins sorting nexin 1 (SNX-1) and SNX-6 (Chen *et al*. 2006). However, the exact mechanisms are not yet completely understood as another study has found that SNX-1 does not influence the recycling of CED-1 to the membrane of the engulfing cell. The levels of CED-1 are controlled by proteosomal degradation. The E3 ubiquitin ligase tripartite motif containing-21 (TRIM-21) was recently found to poly-ubiquitinate CED-1 (Yuan *et al*. 2022). However, the mechanisms guiding CED-1 localization in the plasma membrane, its general internalization, and recycling when not engaged in engulfment are currently unknown.

Here we show that CED-6 acts redundantly with CME in maintaining correct localization of CED-1 on the plasma membrane, as the depletion of *ced-6* together with subunits of AP2 and clathrin results in mislocalization of CED-1 in distinct puncta in the gonadal sheath cells. This control of CED-1 localization occurs independently of the engulfment of the apoptotic cells. The depletion of proteins involved in endocytic sorting and recycling does not affect CED-1 localization in *ced-6* mutants. In agreement with this, we do not observe CED-1::GFP in late (RAB-7-positive) endosomes. Thus, we propose a novel role of CED-6 and CED-7 in maintaining correct localization of CED-1 on the plasma membrane likely via ensuring correct endocytosis of CED-1. Whether CED-6 and CED-7 are more generally involved with endocytosis remains to be shown.

## Materials and methods

### Strains and culture conditions

All strains were maintained at 20°C on standard Nematode Growth Medium (NGM) plates spotted with the *Escherichia coli* strain OP50. The following strains were used: Wild-type N2, linkage group I: VC1026  *rab-10(ok1494)*, linkage group III: *ced-6(tm1826),*  MT4983  *ced-7(n1996),* linkage group IV: MT1522  *ced-3(n717),* linkage group V: MD701 (*bcIs39*[P(*lim-7)ced-1*::GFP + *lin-15*(+)]), DH1201  *rme-1(b1045)*. The *ced-6(tm1826)* mutant was obtained from the National BioResource Project, Japan. The RAB-7::mCherry strain was a kind gift from Me ijiao Li, Xiaochen Lab. All other strains were obtained from the *Caenorhabditis* Genetics Center (CGC), US.

For generation of double and triple mutants, the presence of *bcIs39* in the F2 and F3 generation was scored by GFP expression. The presence of *ced-6(tm1826)*, *rab-10(ok1494)*, and *rme-1(b1045)* was selected by PCR. The presence of *ced-7(n1996)* was selected by the presence of unengulfed apoptotic corpses in 4-fold embryos. Selection for *ced-3(n717)* was done by using the *unc-26(e1196)* mutation as a marker.

### RNAi

The RNAi clones against *dlc-1*(T26A5.9), *tat-1*(Y49E10.11), *sem-5*(C14F5.5), *par-6*(T26E3.3), *gtf-2E2*(F54D5.11), *pkc-3*(F09E5.1), *cdc-42*(R07G3.1), *gtf-2E1*(ZK550.4), *dpy-23*(R160.1), *aps-2*(F02E8.3), *apa-2*(T20B5.1), *apb-1*(Y71H2B.10), *dab-1*(M110.5), *epn-1*(T04C10.2), *dnj-25*(W07A8.3), *dyn-1*(C02C6.1), *itsn-1*(Y116A8C.36), *rab-5*(F26H9.6), *rab-7*(W03C9.3), *rab-10*(T23H2.5), *rab-11.1*(F53G12.1), *rab-35*(Y47D3A.25), *snx-1*(C05D9.1), *snx-6*(Y59A8B.22), *vps-26*(T20D3.7), *arf-6*(Y116A8C.12), and *pld-1*(C04G6.3) were obtained from the OpenBiosystems RNAi Library (Thermo Fisher Scientific, Roskilde, DK, Denmark). The RNAi clones against *dhc-1*(T21E12.4) and *chc-1*(T20G5.1) were from the Ahringer RNAi Library (Source BioScience LifeSciences, Nottingham, UK). RNAi was performed by feeding on NGM plates containing 1 mM isopropyl thiogalactoside and ampicillin (100 *μ*g/ml) as described (Timmons and Fire 1998). Worms fed HT115 bacteria containing an empty pL4440 vector (ctrl RNAi) were used as controls. All assays were performed with worms grown on RNAi for one generation from eggs or L4 when indicated.

### CED-1 localization

Worms were synchronized as eggs and CED-1 localization was scored 96 h later. Worms were cultured at 25°C to obtain stronger expression of CED-1::GFP compared growth at 20°C (Supplementary Fig. 1). They were anesthetized in 25 mM Sodium Azide in S-basal [0.1 M NaCl, 0.05 M H_2_PO_4_ (pH 6)] and mounted on 2% agarose pads.

For visual scoring of CED-1 mislocalization, a Zeiss Axiophot microscope equipped with an Andor Zyla 4.2 sCMOS camera was used. Gonad arms were scored as having CED-1 mislocalized if clear and distinct CED-1::GFP patches were visible. Gonad arms without clear and distinctive patches of CED-1::GFP were scored as having no CED-1 mislocalization. The percentages of gonads with CED-1 mislocalization were calculated and plotted. At least three independent replicates were performed for each experiment.

For higher resolution imaging, the worms were imaged using an Olympus IX83 inverted microscope with a Yokogawa CSU-W1 spinning disk unit equipped with a Hamamatsu Orca-Flash 4.0 camera, 100× as indicated. Z-stacks were acquired and maximum projections over Z images were generated using the Olympus CellSens software.

### Analysis of CED-1::GFP patches

The ilastik pixel classification workflow (Berg *et al*. 2019) was used to characterize CED-1::GFP patches. Representative Z-stack images of *C. elegans* treated with each RNAi clone, including empty vector, were used to train the algorithm in which objects recognized as CED-1::GFP patches were marked separately from background fluorescence. Pixel classification was performed manually on all pictures until a satisfactory and consistent prediction could be made. The batch processing tool was utilized to perform predictions on the remaining data. In Fiji (Schindelin *et al*. 2012), “MaxEntropy” thresholding was performed on the ilastik predictions followed by the “Analyze Particles” function with a size limit range of 35–1,500 pixels^2^ and a circularity limit range of 0.60–1.00. To allow multiple comparisons, an unpaired *t*-test with Bonferroni correction was used to test for significant differences.

### Immunostaining

Immunostaining was performed as previously described (Harders *et al*. 2018). Briefly, worms were transferred to a poly-L-lysine microscopy slide (VWR, Denmark); the cuticle was punctured with a sharp needle to allow for better exposure of the germline. Freeze-cracking to rip off the cuticle was performed by gently placing a coverslip on top followed by snap freezing at −80°C. After the slide was fully frozen, the coverslip was removed with a scalpel. The slides were incubated 30 min in ice-cold methanol and washed in PBS. Worms were encircled with a PAP pen (ThermoFischer) and then blocked 2 h in 2% (*w*/*v*) milk-PBS before overnight incubation with primary antibodies against GFP (A-11122, Invitrogen), Clathrin/CHC-1 (610500, BD Transduction Laboratories) and Dynamin/DYN-1 (DYN1, DSHB). The dilutions used were 1:2,000, 1:125, and 1:20 (from a 0.06 mg/mL self-purified stock), respectively, in 2% milk-PBS. After incubation, the slides were washed once in PBS and incubated for 2 h with a goat antimouse Alexa488 conjugated antibody (Life Technologies, Denmark) and a goat antirabbit Cy5 conjugated antibody, diluted 1:2,000 and 1:1,000, respectively, in 2% milk-PBS. The slides were washed once in Tris-Buffered Saline, 0.1% Tween 20 Detergent (TBST) followed by 2 times in PBS, fixed 15 min in 2% PFA, and mounted with Fluoromount-G Mounting Medium (Invitrogen) before spinning disk confocal imaging.

### Western blotting

The samples for Western Blot were prepared by adding 20 worms to 20 µL S-basal and snap-freezing in liquid nitrogen before performing SDS-PAGE. The ladder used was iBright Prestained Protein Ladder (LC5615, Invitrogen). The protein bands on the gel were transferred to a nitrocellulose membrane using Trans-Blot Turbo system (Bio-Rad). Blocking was performed for 2 h at room temperature in 2% milk-TBST, followed by overnight incubation at 4°C with the primary antibodies anti-Clathrin/CHC-1 (610500, BD Transduction Laboratories) and anti-Dynamin/DYN-1 (DYN1, DSHB). The dilutions used were 1:250 and 1:60 (from a 0.06 mg/mL self-purified stock) in 2% milk-TBST, respectively. The membrane was washed once with TBST before incubating with secondary antibody anti-Mouse IgG (Fc specific) Highly-X-Adsorbed-HRP (SAB3701029-500 *µ*g, Sigma-Aldrich) for 1–2 h at room temperature using a 1:1,000 dilution. The membrane was then washed 3 times with TBST and visualized using Amersham ECL Prime western blotting detection reagent (GERPN2232, Cytiva).

## Results

Previously, we and others have shown that dynein light chain 1, *dlc-1*, plays several roles in apoptosis (Morthorst and Olsen 2013; Harders *et al*. 2018; Zhang *et al*. 2022). For the present study, we constructed a strain expressing the CED-1::GFP marker in a *ced-6* mutant background to monitor the restoration of the engulfment. The CED-1::GFP fusion protein has previously been shown to be functional and capable of rescuing *ced-1* mutants (Zhou *et al*. 2001).

**Fig. 1.**
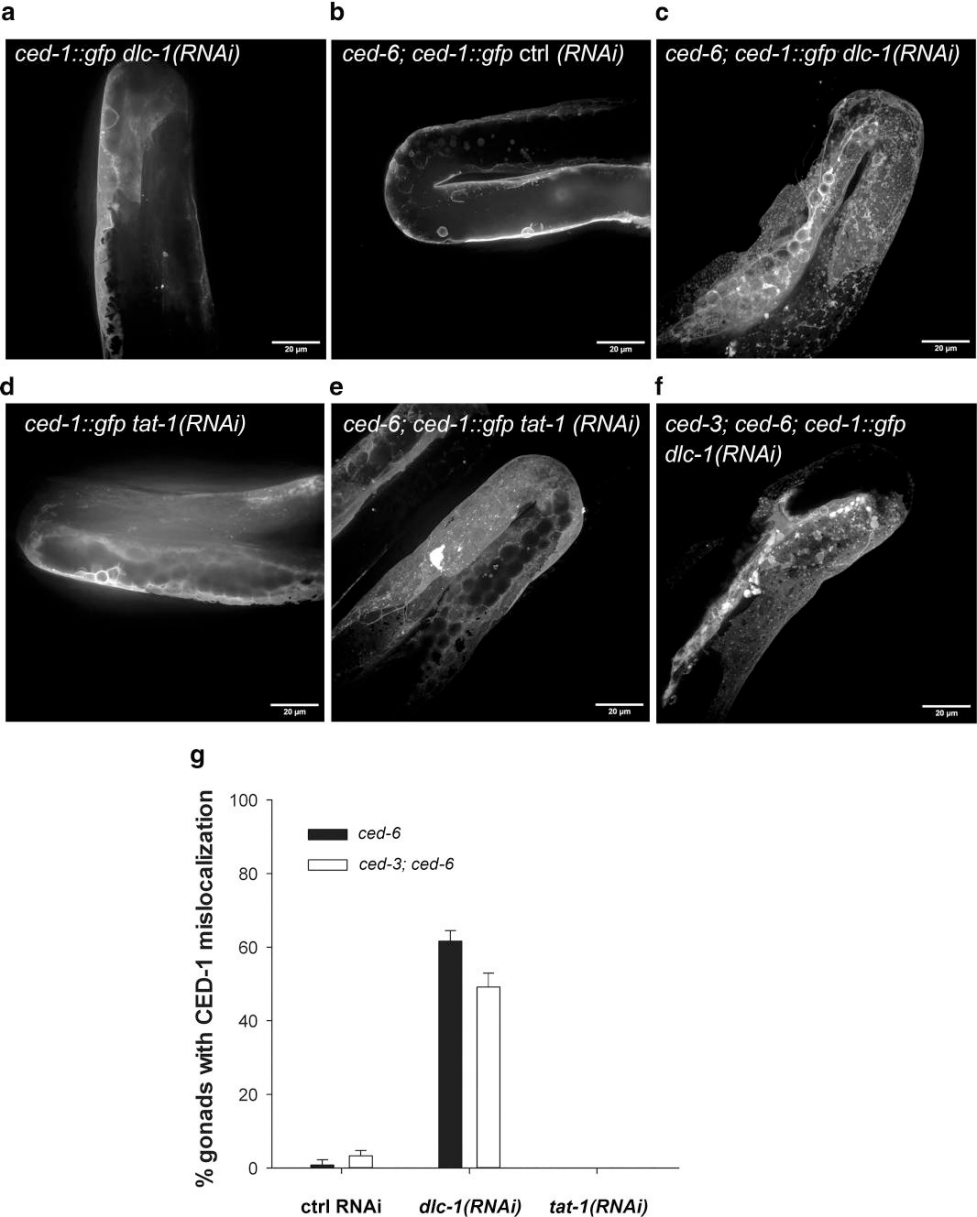
Simultaneous loss of *dlc-1* and *ced-6* causes mislocalization of CED-1. a) Normal CED-1 localization in *ced-1::gfp; dlc-1(RNAi).* b) Normal CED-1 localization in *ced-6*; *ced-1::gfp* animals. c) Mislocalization of CED-1 in *ced-6*; *ced-1::gfp* animals treated with RNAi against *dlc-1*. d) Normal CED-1 localization in *ced-1::gfp* treated with RNAi against *tat-1*. e) Normal CED-1 localization in *ced-6*; *ced-1::gfp* treated with RNAi against *tat-1* f) Mislocalization of CED-1 in *ced-3*; *ced-6; ced-1::gfp* animals fed RNAi against *dlc-1*. g) Quantification of gonads with mislocalized CED-1 in *ced-6*; *ced-1::gfp* and *ced-3; ced-6; ced-1::gfp* animals treated with empty vector control RNAi or RNAi against *dlc-1* or *tat-1*. Mean of at least three independent experiments ± SD. *N* = 40 in each experiment.

In agreement with previous studies (Zhou *et al*. 2001), we found that the CED-1::GFP fusion protein is expressed in sheath cells where it has an even distribution (Fig. 1a). Interestingly, RNAi knockdown of *dlc-1* in this *ced-6* mutant background caused mislocalization of CED-1. The removal of *dlc-1* by means of RNAi did not have an effect on CED-1::GFP localization, which remained evenly distributed in the sheath cells (Fig. 1a) when CED-6 was present. Likewise, mutation of *ced-6* did not affect CED-1::GFP localization (Fig. 1b) when DLC-1 was present. However, following simultaneous removal of *dlc-1* and *ced-6*, CED-1::GFP localized in specific puncta in the sheath cells (Fig. 1c), very distinctively from the even distribution in controls. Thus, CED-6 and DLC-1 have redundant functions in ensuring correct localization of CED-1.

DLC-1 has both dynein-dependent and -independent functions (Rapali *et al*. 2011; Ellenbecker *et al*. 2019; Day *et al*. 2022; Fielder *et al*. 2022). To investigate whether DLC-1 acts as a part of the dynein complex, we used RNAi against the dynein heavy chain, *dhc-1*, to test whether this could phenocopy RNAi against *dlc-1*. The knockdown of *dhc-1* in *ced-6*;*ced-1::gfp* animals did not result in CED-1 mislocalization (Table 1), indicating that DLC-1 regulates CED-1 localization independently of its association with the dynein complex. The knockdown of *dhc-1* from eggs resulted in larval arrest (data not shown) verifying that the *dhc-1* RNAi clone was effective.

**Table 1.** List of genes tested for effects on CED-1 localization.

Gene	% mislocalization in ced-1::gfp	% mislocalization in ced-6; ced-1::gfp	Phenotype in Balklava *et al*. (2007)	Endocytic compartment
**EV**	1.9 ± 1.9	2.2 ± 2.2	—	—
**dlc-1**	N.D.	61.7 ± 2.8	weak	—
**dhc-1**	0.0	1.7 ± 1.4	N.I.	—
***chc-1***	32.9 ± 5.1	47.8 ± 10.5	Strong	CME
***dpy-23***	1.9 ± 1.9	74.4 ± 19.2	N.I.	CME
***aps-2***	1.7 ± 1.7	71.9 ± 21.3	N.I.	CME
***apa-2***	3.1 ± 3.1	45.6 ± 33.0	N.I.	CME
***apb-1***	0.6 ± 0.6	30.4 ± 23.2	N.I.	CME
***dab-1***	0.6 ± 0.6	2.6 ± 2.6	strong	CME
***epn-1***	10.5 ± 7.1	25.1 ± 15.8	weak	CME
***dnj-25***	6.8 ± 4.1	16.0 ± 2.4	N.I.	CME
***dyn-1***	N.D.	89.6 ± 10.0	strong	CME
***itsn-1***	N.D.	24.6 ± 5.6	N.I.	CME
***rab-5***	62.1 ± 12.6	91.3 ± 7.8	strong	EE
***rab-7***	0.0	14.7 ± 14.7	N.I.	LE
***rab-10***	N.D.	4.8 ± 4.8	weak	EE to TGN
***rab-11.1***	0.9 ± 0.9	5.0 ± 5.0	N.I.	ERC/TGN
***rab-35***	N.D.	6.8 ± 3.4	N.I.	RRC
***snx-1***	N.D.	24.2 ± 24.2	N.I.	E to TGN
***snx-6***	N.D.	8.0 ± 5.1	N.I.	E to TGN
***arf-6***	N.D.	7.3 ± 6.9	N.I.	RE
rme-1*	15.0 ± 5.1	16.5 ± 11.4	N.I.	RE
***vps-26***	N.D.	6.4 ± 6.3	N.I.	E to TGN
rab-10*	N.D.	0.0	N.I.	EE to ERC

Mean of at least 3 independent experiments ± SD. *N* = 20–40 in each experiment.

N.I., not identified; N.D., not determined; CME, clathrin-mediated endocytosis; E, endosomes; EE, early endosomes; LE, late endosomes; ERC, endocytic recycling compartment; E-TGN, endosomes to Trans-Golgi network; RE, recycling endosomes; RRC, rapid recycling compartment. *Mutant tested; otherwise, RNAi was used.

TAT-1 (Transbilayer Amphipath Transporter) is a membrane translocase that keeps the “eat-me” signal phosphaditylserine (PS) on the inner leaflet of the plasma membrane (Darland-Ransom *et al*. 2008; Chen *et al*. 2019). To test if incorrect exposure of “eat-me” signals caused the CED-1 puncta, we took advantage of *tat-1* RNAi, which causes ectopic exposure of PS (Darland-Ransom *et al*. 2008). Knockdown of *tat-1* did not alter CED-1 distribution in wild-types nor in in *ced-6*;*ced-1::gfp* mutants (Fig. 1, d, e, and g). This is consistent with the puncta being inside the sheath cells and not bound to eat-me signals.

Next, we investigated if the CED-1 puncta were associated with the recognition of apoptotic cells. To this end, we constructed a *ced-3*;*ced-6*;*ced-1::gfp* strain. *ced-3* encodes a caspase necessary for the most common forms of apoptosis in *C. elegans* and mutation of *ced-3* completely blocks apoptosis. We found that lack of apoptosis did not alter the presence of CED-1 puncta in *ced-3*; *ced-6*, *dlc-1(RNAi)* animals (Fig. 1, f and g). Thus, the localization of CED-1 is altered independently of induction of apoptosis and engulfment. Therefore, we conclude that the CED-1 puncta are not due to CED-1 binding to signals on the surface of apoptotic cells, but rather CED-1 puncta are formed inside the sheath cells.

**Fig. 2.**
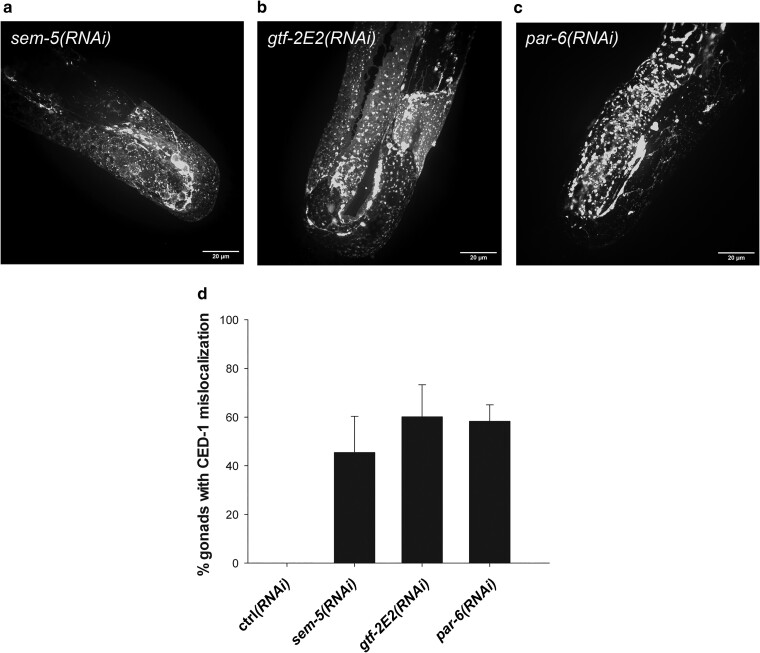
Screen for regulators of CED-1 localization. RNAi against *sem-5* (a), *gtf-2E2* (b), or *par-6* (c) causes mislocalization of CED-1 in *ced-6*; *ced-1::gfp* animals. d) Quantification of gonads with mislocalized CED-1 in *ced-6*; *ced-1::gfp* animals treated with RNAi against *sem-5*, *gtf-2E2*, and *par-6*. Mean of at least 3 independent experiments ± SD. *N* = 40 in each experiment.

**Fig. 3.**
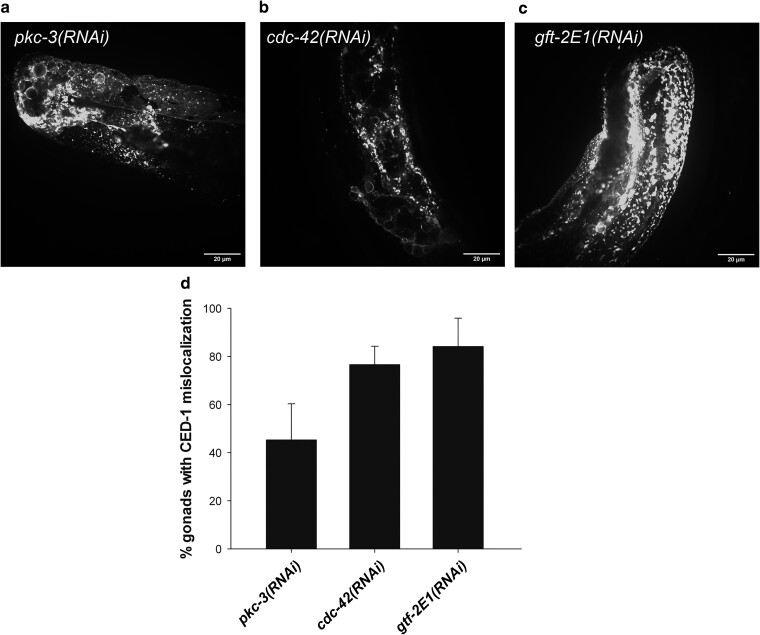
Analysis of candidate genes causing CED-1 mislocalization. RNAi against pkc-3 (a), *cdc-42* (b), or *gtf-2E1* (c) causes mislocalization of CED-1 in *ced-6*; *ced-1::gfp* animals. d) Quantification of gonads with mislocalized CED-1 in *ced-6*; *ced-1::gfp* animals treated with RNAi against *pkc-3*, *cdc-42*, and *gtf-2E1*. Mean of at least 3 independent experiments ± SD. *N* = 40 in each experiment.

To further our mechanistic understanding of the CED-1 mislocalization, we performed an RNAi screen (Fig. 2) to identify additional genes that upon knock down influence CED-1 localization. We used the *ced-6*;*ced-1::gfp* strain and screened the first ∼1,300 clones of the Open Biosystem RNAi library. In the initial screen we identified 11 clones, which caused mislocalization of CED-1::GFP in a *ced-6* mutant background. Upon several rounds of re-testing three clones, *sem-5*, *par-6*, and *gtf-2E2* were found to reproducibly cause CED-1 mislocalization (Fig. 2, a–d). The gene *sem-5* encodes an Src homology domains 2 and 3 protein, orthologous to the human growth factor receptor-bound protein 2 (Clark *et al*. 1992; Lowenstein *et al*. 1992). SEM-5 acts downstream of the epidermal growth factor receptor LET-23 and functions in multiple signaling pathways during development and embryogenesis (Hopper *et al*. 2000; Worby and Margolis 2000). The gene *par-6* encodes a PDZ-domain-containing protein with close homology to the human PAR-6G and is involved in the regulation of cell polarity (Hung and Kemphues 1999; Nance *et al*. 2003) and organization of noncentrosomal microtubules (Castiglioni *et al*. 2020). GTF-2E2 encodes an ortholog of the General transcription factor IIE beta (TFIIE β) subunit and is involved with initiation of transcription (Yamamoto *et al*. 2001). To elaborate our findings, we tested the interaction partners of our identified genes for induction of the CED-1 phenotype. GTF-2E2 interacts with GTF-2E1, the TFIIE alpha subunit ortholog. PAR-6 forms a complex with PAR-3, PKC-3, and CDC-42 (the PAR complex) to establish embryonic and epithelial polarity (Izumi *et al*. 1998; Nance *et al*. 2003; Nance 2005; Li *et al*. 2010). We treated *ced-6*;*ced-1::gfp* animals with RNAi against *gtf-2E1*, *pkc-3*, and *cdc-42*. The knockdown of all three genes caused CED-1 mislocalization similar to their interaction partners (Fig. 3, a–d), confirming that our screen was able to identify new regulators of CED-1 localization. Strikingly, all the genes identified in our screen and their tested interaction partners were also identified in a screen for proteins involved in yolk uptake via receptor mediated endocytosis by oocytes (Balklava *et al*. 2007). Furthermore, PAR-6 and CDC-42 affect clathrin, encoded by *chc-1* in *C. elegans* (Balklava *et al*. 2007). Hence, we turned to investigate the role of CME in CED-1 localization.

**Fig. 4.**
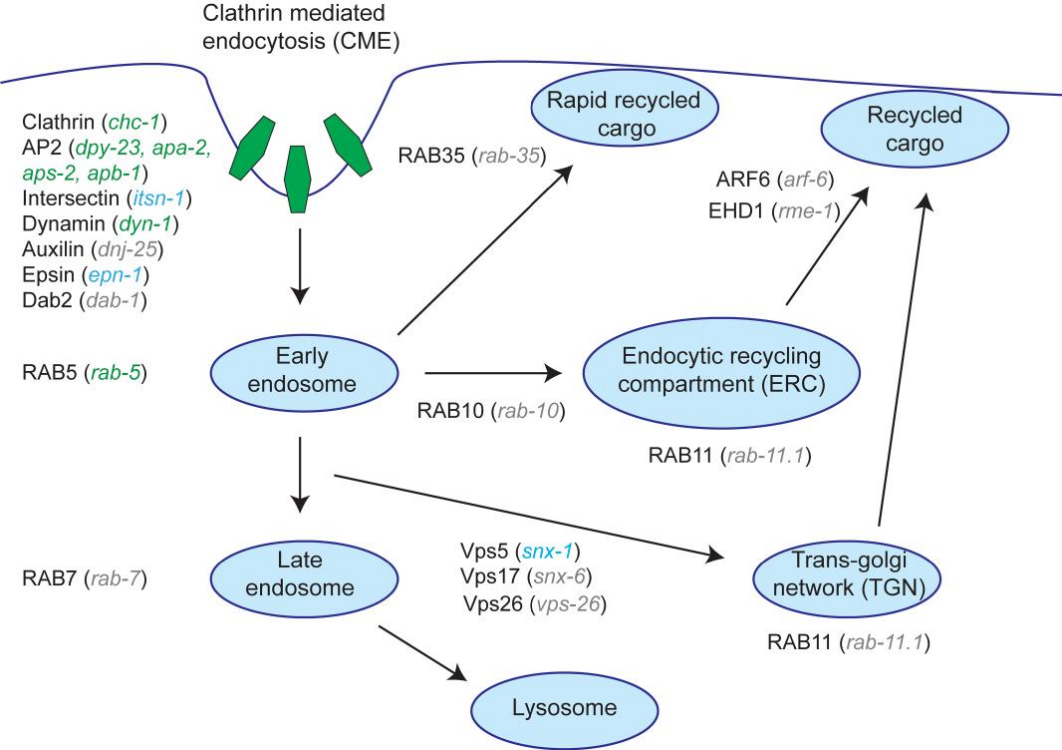
Genes involved with CME and endocytic recycling. Green: genes affecting CED-1 localization, blue: genes having a slight effect on CED-1 localization, gray: genes with no effect on CED-1 localization.

**Fig. 5.**
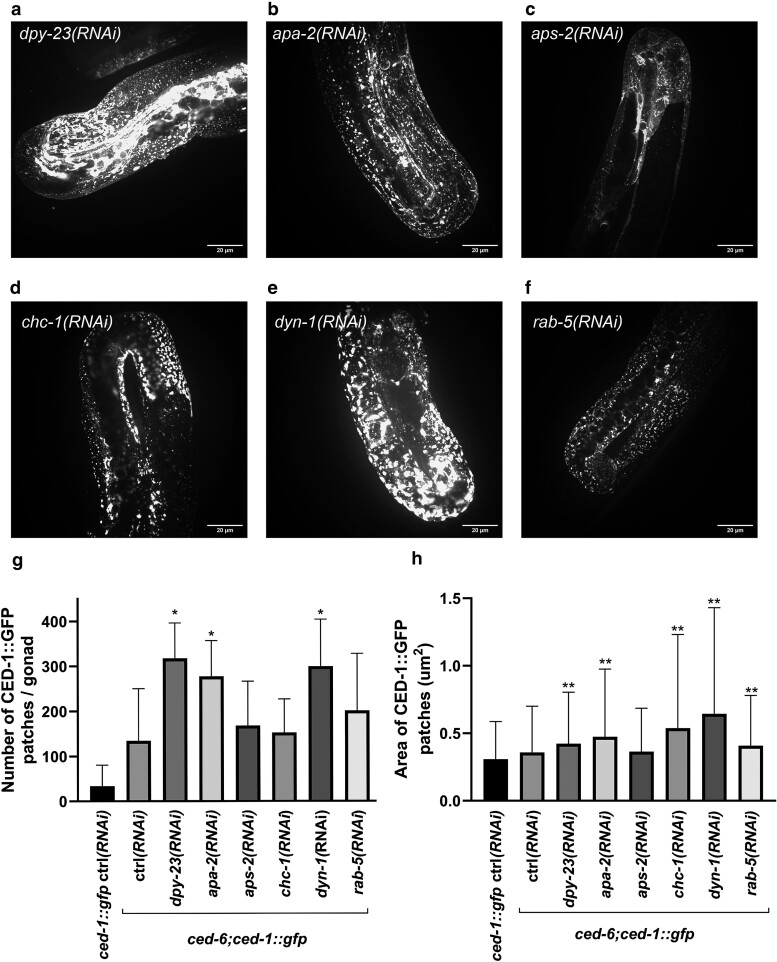
Proteins involved in CME act redundantly with CED-6 in CED-1 localization. RNAi against *dpy-23* (a), *apa-2* (b), *aps-2* (c), *chc-1* (d), *dyn-1* (e), or *rab-5* (f) causes mislocalization of CED-1 in *ced-6*; *ced-1::gfp* animals. RNAi against *chc-1* or *dyn-1* was introduced on L4 animals. g) Number of CED-1::GFP patches in *ced-6*, *ced-1::gfp*, and *ced-1::gfp* animals treated with RNAi against CME-1 related genes and empty vector control RNAi. *N* = 6–45. *P-value< 0.05. h) Area of CED-1 patches (µm^2^) in *ced-6*, *ced-1::gfp*, and *ced-1::gfp* animals with RNAi against CME-related genes and empty vector control RNAi. *N* = 499–6,050. **P-value < 0.001.

Transmembrane receptors are continuously being internalized and either degraded or recycled to the cell surface. The molecular mechanisms of CED-1 recycling when it is not engaged in engulfment are largely unknown. We hypothesize that the CED-1 puncta caused by simultaneous knockdown of *ced-6* and *dlc-1* result from a defect either in CED-1 internalization or from defective endocytic sorting and recycling. To test this, we genetically dissected the known endocytosis and recycling pathways. We chose a panel of genes (Fig. 4) covering CME, early endosomes, late endosomes, different pathways for endocytic recycling, and the trans-Golgi network to investigate their effect on CED-1 localization in the *ced-6* mutant background. To study CME, we chose the clathrin heavy chain, *chc-1*, and the 4 subunits of the AP2 adaptor complex, *apa-2* (α subunit), *apb-1* (β2 subunit), *dpy-23* (µ2 subunit), and *aps-2* (σ2 subunit). We knocked down these genes by RNAi in *ced-6; ced-1::gfp* animals and scored them for the presence of CED-1 mislocalization. RNAi against *chc-1*, *dpy-23*, *apa-2, and aps-2* robustly produced the CED-1 mislocalization, while knockdown of *abp-1* resulted in lesser penetrating phenotypes than the rest (Fig. 5, a–d and Table 1). This result demonstrates that CME is involved in correct localization of CED-1 together with CED-6. CHC-1 and AP2 mediate the engulfment of apoptotic cells downstream of the CED-1 pathway (Chen *et al*. 2013). To further strengthen our observation that the observed CED-1 mislocalization occurs independently of its role in engulfment, we knocked down *chc-1* in a *ced-3; ced-6* mutant background to eliminate all apoptotic and engulfment events. Like RNAi against *dlc-1*, the knockdown of *chc-1* caused the same degree of CED-1 puncta in the *ced-3* background (data not shown), demonstrating that this phenotype is apoptosis and engulfment independent.

Several other proteins besides clathrin and AP2 regulate CME. The gene *dab-1* encodes a homolog of mammalian ARH and Dab2, and it is essential for low-density lipoprotein receptor (LDLR) endocytosis (Garcia *et al*. 2001). The gene *epn-1* encodes epsin (Chen *et al*. 1998), which binds clathrin and coordinates endocytosis of specific receptors (Maldonado-Báez and Wendland 2006). The gene *dnj-25* encodes the homolog of auxilin, which is involved in uncoating of clathrin-coated vesicles (Massol *et al*. 2006; Taylor *et al*. 2011). Dynamin, *dyn-1*, is required for vesicle budding and scission (Hinshaw and Schmid 1995; Sweitzer and Hinshaw 1998; Ferguson and De Camilli 2012), and *itsn-1* encodes intersectin, which functions in nucleation of clathrin-coated pits (Henne *et al*. 2010; Herrero-Garcia and O’Bryan 2017). To identify if additional proteins of CME affect the localization of CED-1, we treated *ced-6; ced-1::gfp* animals with RNAi against these genes. Surprisingly, it was only RNAi against *dyn-1* that strongly displayed CED-1 mislocalization (Fig. 5e and Table 1). RNAi against *dab-1* and *dnj-25* had no apparent effect, and RNAi against *epn-1* and *itsn-1* produced only a weak effect on CED-1 localization (Table 1). In conclusion, correct CED-1 localization depends on the redundant actions of CED-6, clathrin, AP2, and dynamin. The other CME regulators either play minor roles or do not seem to be involved with CED-1 localization, validating that CME utilizes different proteins depending on which receptors it is internalizing.

To investigate whether the CED-1 mislocalization could also result from blocking endocytic recycling, we knocked down genes known to function downstream of CME in endocytic sorting and recycling in a *ced-6* mutant background and tested whether they phenocopied the knockdown of clathrin and subunits of the AP2 complex. RAB-5 is a marker for early endosomes, while RAB-7 associates with late endosomes (Kinchen *et al*. 2008; Yu *et al*. 2008; Poteryaev *et al*. 2010) as well as endocytic recycling compartments (ERCs) (Harrison *et al*. 2014). RAB-10 is necessary for the progression of early endosomes to ERC (Babbey *et al*. 2006; Chen *et al*. 2006). RAB-11 localizes to ERC and the GTGN (Weigert *et al*. 2004; Winter *et al*. 2012). The retromer complex subunits SNX-1, SNX-6, and VPS-26 are involved in recycling to TGN or the plasma membrane and have been shown to mediate the recycling of CED-1 back to the plasma membrane after engulfment of apoptotic cells (Chen *et al*. 2010). RAB-35 mediates rapid recycling, while ARF-6 mediates slower recycling from the ERC (Radhakrishna and Donaldson 1997; Jovanovic *et al*. 2006; Kouranti *et al*. 2006; Sato *et al*. 2008). RNAi against *rab-5* in *ced-6; ced-1::gfp* animals was the only gene to produce a strong phenotype of mislocalized CED-1 (Fig. 5f and Table 1). RNAi against *snx-1* resulted in a mild CED-1 mislocalization phenotype, while RNAi against the rest of the genes had no effect on CED-1 localization (Table 1). This suggests that inhibition of endocytic sorting and recycling in general does not interfere with CED-1 localization.

We were expecting both endocytic sorting and recycling would play a role in localizing CED-1 at the plasma membrane. Hence, we were intrigued that SNX-1 was the only recycling factor that upon knockdown caused CED-1 mislocalization and that the effect was not particularly strong.

To rule out that the lack of effect was due to inefficient RNAi, we tested true mutants of *rab-10(ok1494)* and *rme-1(b1045)*. RAB-10 is required for the progression of early endosomes to ERC (Babbey *et al*. 2006; Chen *et al*. 2006) and RME-1 is a conserved recycling factor functioning in late steps of endocytic recycling (Grant and Caplan 2008; Grant and Donaldson 2009). In *rab-10; ced-6* double mutants, we did not observe any CED-1 mislocalization, and in *rme-1; ced-6* double mutants, we only saw a small increase (<15%) in CED-1 mislocalization. Interestingly, CED-1 mislocalization in *rme-1* mutants was observed even if CED-6 was present (Table 1). This suggests that impaired recycling per se is not the causal component in CED-1 mislocalization, rather earlier steps in CME appear to be important. Consistent with this hypothesis, depletion of *rab-5* causes CED-1 puncta similar to other genes important for the steps of CME. RAB-5 is required for efficient uncoating and associates with clathrin-coated vesicles at an early stage (Semerdjieva *et al*. 2008). Interestingly, we find that RNAi against *chc-1* and *rab-5* in a wild-type background cause mislocalization of CED-1 (Table 1), albeit to a lesser extent than in the *ced-6* mutant background. This suggests that CHC-1 and RAB-5 are only partially redundant with CED-6. This is consistent with CHC-1 and RAB-5 being the main proteins required for CME and early endosome dynamics.

We observed that the CED-1 puncta had different appearances following RNAi-mediated knockdown of the tested genes (Fig. 5, a–f). This was supported by the quantification of the number and sizes of the CED-1 puncta (Fig. 5, g and h). For instance, RNAi of *dyn-1* causes the largest CED-1::GFP puncta, whereas RNAi of *dpy-23* displays the highest number of CED-1::GFP patches. It is possible that the patches of different sizes simply represent different quantities of mislocalized CED-1::GFP. However, the different appearances could also be the result of retention in different subcellular compartments/vesicles.

**Fig. 6.**
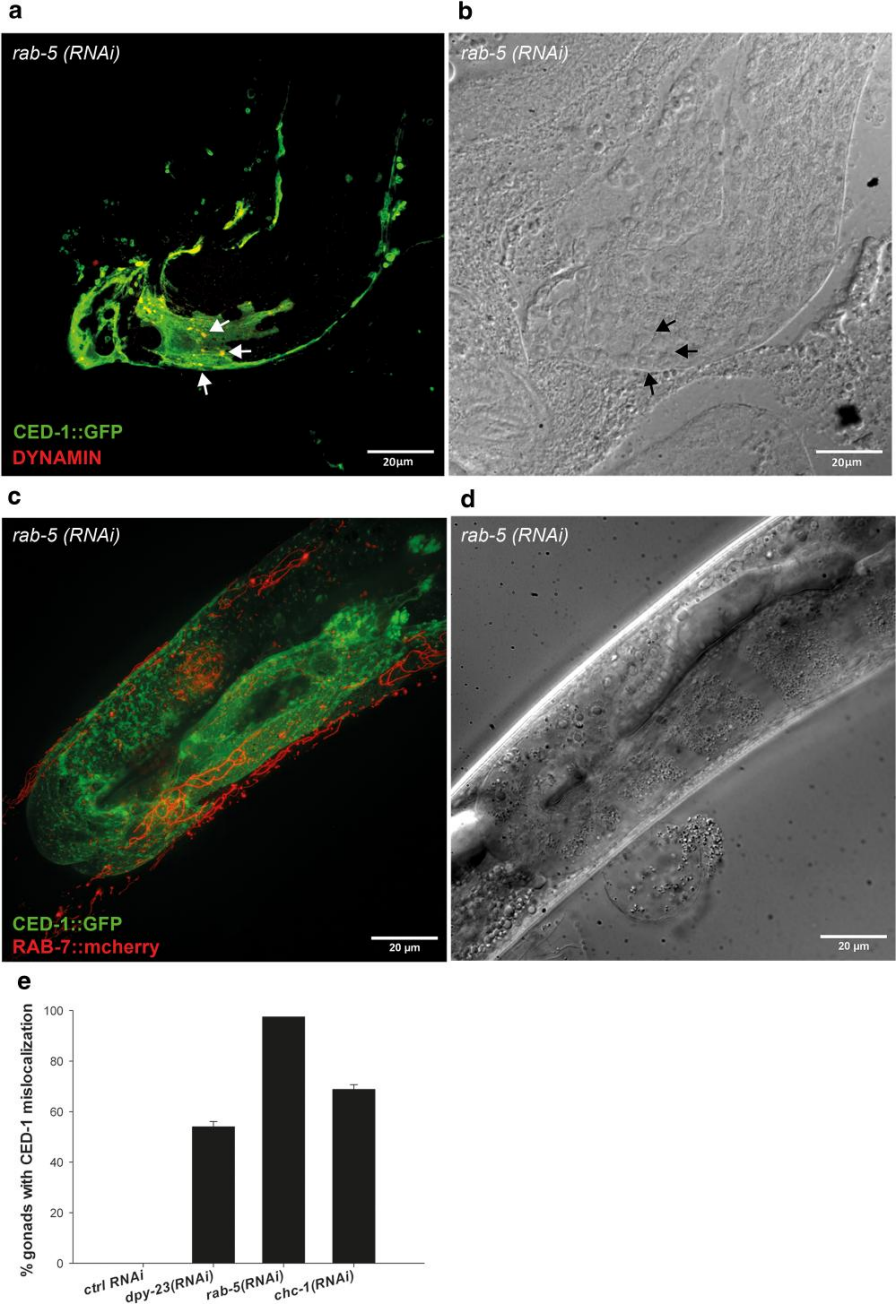
CED-1 mislocalization colocalizes with the early CME marker DYN-1 and CED-7 functions together with CED-6 to mediate CED-1 localization. a) Fluorescence image of CED-1::GFP patches in *ced-6*; *ced-1::gfp* animals treated with RNAi against *rab-5* that colocalize with dynamin stained with *anti-dyn-1*. b) Corresponding DIC image to 6A. c) Image of CED-1::GFP puncta in *ced-6*; *ced-1::gfp* animals treated with RNAi against *rab-5*. CED-1::GFP puncta do not colocalize with RAB-7::mCherry positive late endosomes. d) Corresponding DIC image to 6C. e) Quantification of gonads with mislocalized CED-1::GFP in *ced-7; ced-1::gfp* animals treated with RNAi against *dpy-23*, *rab-5*, and *chc-1*. Mean of at least 3 independent experiments ± SD. *N* = 20–40 in each experiment.

A prediction of our data is that CED-1 should be retained at or near the plasma membrane. To test this, we performed colocalization studies with DYN-1 and CHC-1. We used antibodies against GFP and DYN-1 and CHC-1, to detect CED-1::GFP and DYN-1 and CHC-1, respectively. To allow access of the antibodies to the sheath cells, worms were subjected to freeze-cracking. Inherently, the freeze-cracking results in heterogeneous samples as the degree of tearing cannot be controlled. However, we found that CED-1::GFP patches colocalized with both early CME markers (Fig. 6, a and b, Supplementary Figs. 2 and 3).

To rule out that puncta represent CED-1 trapped in late endosome compartments, we assayed for colocalization between CED-1::GFP puncta and RAB-7 fused to mCherry (Li *et al*. 2009). The CED-1::GFP puncta did not colocalize with RAB-7::mCherry (Fig. 6, c and d). CED-6 and CED-7 act in a common genetic pathway to regulate engulfment of apoptotic cells through CED-1 (Zhou *et al*. 2001). In fact, CED-7 acts both upstream (dying cell) and downstream (engulfing cell) of CED-1 and is required for CED-1 to recognize apoptotic cells, and CED-6 functions downstream of CED-1 (Zhou *et al*. 2001). We therefore hypothesized that lack of CED-7 would also cause mislocalization of CED-1 following inactivation of genes involved with receptor internalization similar to CED-6. To test this, we constructed a *ced-7; ced-1::gfp* strain and knocked down *chc-1*, *dpy-23*, and *rab-5* with RNAi. All three genes showed CED-1 mislocalization to a similar degree as in the *ced-6* mutant background (Fig. 6e), confirming that CED-7 acts together with CED-6 to mediate correct localization of CED-1.

## Discussion

CED-1 is a transmembrane receptor involved in recognition of “eat-me” signals on apoptotic cells guided by CED-7. The subsequent engulfment is mediated by CED-6. The downstream engulfment pathway from CED-1 and CED-6 is extensively characterized. In this study, we have shown that lack of *ced-6* or *ced-7* in itself does not affect the localization of CED-1. However, when they are depleted together with either *chc-1* or the subunits of the AP2 complex, which regulate CME, it causes mislocalization of CED-1 in distinct puncta.

To obtain strong expression of the CED-1::GFP reporter, all experiments were performed at 25°C. Compared with the culture at 20°C, a higher temperature can influence aggregation of fusion proteins (Cahoon *et al*. 2023). However, we also observed CED-1::GFP puncta when worms were cultured at 20°C. Since all comparisons are made between worms cultured at 25°C, the differences in CED-1 mislocalization are indeed due to the RNAi-mediated knockdown of the investigated genes. Supporting this view, we find that depending on the genes inactivated, the CED-1 patches have different appearances. This variation likely results from different levels of retained CED-1::GFP rather than different localizations since RNAi against *chc-1*, *apa-2*, and *rab-5* all colocalize with the early CME markers DYN-1 and CHC-1.

**Fig. 7.**
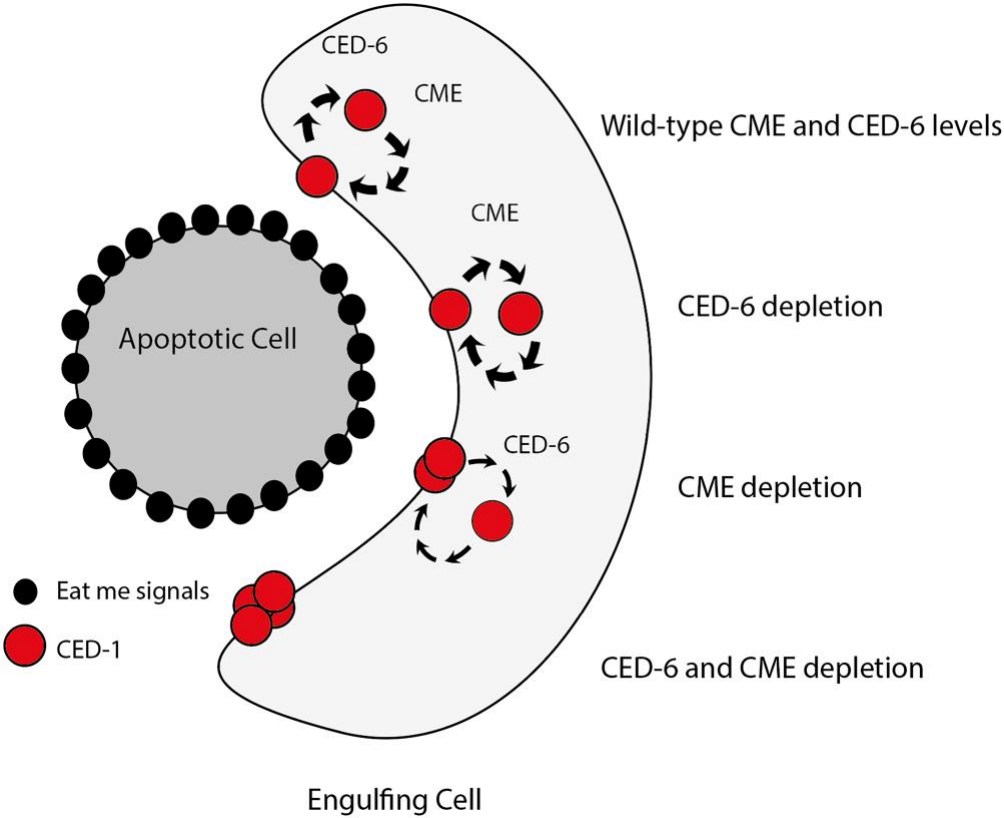
CED-6 and CME components act redundantly to correctly display CED-1. Proposed model for CED-6 and CME components processing CED-1 in wild-types, *ced-6* mutants, reduced CME, and finally simultaneous loss of CED-6 and CME. For clarity, the 4 scenarios and their consequences on CED-1 localization are shown in the same engulfing cell.

Our findings can be explained by a model where the genes *ced-6* and *ced-7* are directly involved with CME of CED-1 and act redundantly with clathrin and the AP2 complex to ensure CED-1 localization on the plasma membrane (Fig. 7). Alternatively, *ced-6* and *ced-7* could be indirectly involved in the recycling of CED-1, for example by changing the recognition of CED-1 by the endocytic proteins. When *ced-6* and/or *ced-7* are present, CME is very effective and hence not sensitive to reductions in endocytic capacity. However, in the absence of *ced-6* or *ced-7*, the CME of CED-1 is impaired, and consequently the recycling process becomes hypersensitive to reductions in endocytic capacity. Finally, we cannot rule out yet other explanations, for example that a block of CME due to lack of CED-6 and AP-2 could in fact be stimulating clathrin-independent endocytosis.

We saw no effect on CED-1 localization when depleting proteins involved in endosome sorting and recycling, demonstrating that inhibition of CME and internalization of CED-1 most likely causes CED-1 to be mislocalized. Many of the proteins we identified to regulate CED-1 localization have known functions in engulfment of apoptotic cells. Our study identifies novel engulfment independent roles for CED-6 and CED-7, as complete inhibition of apoptosis, and hence engulfment, by mutation of the CED-3 caspase, still caused CED-1 mislocalization in the various backgrounds tested. Additionally, ectopic expression of the PS eat-me signal by the knockdown of *tat-1*, which would stimulate the recognition of cells by CED-1, did not affect CED-1 localization, further stressing that the CED-1 phenotype is engulfment independent. During the engulfment of apoptotic cells, CED-6 and CED-7 are considered to act in the same pathway (Lettre and Hengartner 2006). It remains to be shown if that is also the case for their involvement in CED-1::GFP localization.

Interestingly, *dlc-1* was isolated in a screen for regulators of yolk uptake in the oocytes (Balklava *et al*. 2007). This yolk uptake screen did not identify *dhc-1*, which strengthens the notion that *dlc-1* acts in endocytosis independently of dynein. Four of the endocytosis genes we tested and found to regulate CED-1 localization were also identified in the yolk uptake screen, namely *chc-1*, *epn-1*, *dyn-1*, and *rab-5* (Table 1). The AP2 subunits did not come up in the yolk screen, but they were identified in an earlier RNAi screen for endocytosis of the yolk (Grant and Hirsh 1999). Interestingly, some of the genes we found not to regulate CED-1 localization were found to mediate yolk uptake (Balklava *et al*. 2007) (Table 1). This illustrates that internalization and recycling are complex processes and that the proteins involved are cargo specific.

### CED-6 is important for CED-1 internalization

The internalization of a receptor depends on sorting signals in its cytoplasmic domain, which are recognized by adaptor proteins like AP2 or CLASPs. The µ2 subunit of AP2 binds to YXXØ sorting signals (Owen and Evans 1998; Olusanya *et al*. 2001), while the σ2 subunit recognizes acidic dileucine sequences, (D/E)XXXL(L/I) (Bonifacino and Traub 2003; Janvier *et al*. 2003; Kelly *et al*. 2008). (F/Y)XNPX(Y/F) signals are generally recognized by PTB domain-containing proteins (Traub 2009). CED-1 contains a NPXY, ^960^FQNPLY, two YXXØ sorting signals, ^1019^YASL and ^1086^YADI (Zhou *et al*. 2001; Su *et al*. 2002), and three potential dileucine-like signals, ^915^LL, 993LL, ^1053^LL. This type of dileucine signal was found in the *Drosophila* vitellogenin receptor Yolkless, where it acts as an internalization signal, probably by binding to AP2 (Jha *et al*. 2012). CED-6 contains a PTB domain and binds directly to the NPXY domain of CED-1, and this interaction is important for transmitting engulfment signals (Zhou *et al*. 2001; Su *et al*. 2002). We propose that this interaction between CED-1 and CED-6 through the recognition of the NPXY sorting signal is important for correct localization of CED-1 as well, and that CED-6 functions in clathrin-mediated internalization of CED-1. This is supported by the *Drosophila* study showing that CED-6 binds to an NPXY signal in the Yolkless receptor and promotes its internalization through CME (Jha *et al*. 2012). Furthermore, CED-6 also promotes engulfment of apoptotic cells in *Drosophila* (Awasaki *et al*. 2006; MacDonald *et al*. 2006; Cuttell *et al*. 2008), and mammalian GULP interacts through its PTB domain with LDL receptor-related protein 1 (LRP1) (Kiss *et al*. 2006), stabilin-2 (Park *et al*. 2008), and amyloid precursor protein (Hao *et al*. 2011; Beyer *et al*. 2012), all of which are transmembrane proteins known to undergo CME (Li *et al*. 2001; Thinakaran and Koo 2008). *C. elegans*  CED-6 interacts directly with clathrin heavy chain, CHC-1, and the α, β2, and µ2 subunits (the σ2 subunit was not tested) of AP2 (Chen *et al*. 2013). Mammalian GULP and fly-CED-6 also binds directly to clathrin and AP2 (Martins-Silva *et al*. 2006; Jha *et al*. 2012), and GULP localizes to clathrin-coated structures and vesicles (Kiss *et al*. 2006; Beyer *et al*. 2012; Vandré *et al*. 2012). These studies further stress the point that CED-6 functions during engulfment and also impacts CME. When simply observed under the microscope, we did not observe mislocalization of CED-1 when only *ced-6* was mutated, other adaptor proteins needed to be depleted together with *ced-6* to see the phenotype. However, when using the more sensitive spinning disk confocal microscopy followed by machine learning quantification, CED-1::GFP patches were detected in *ced-6* mutants albeit to a much lower degree compared with the simultaneous inactivation of *ced-6* in CME components. This suggests that two redundant pathways regulate the localization and internalization of CED-1, or that the mutation of CED-6 sensitizes the CME machinery.

### Receptor internalization requires multiple sorting signals and adaptor proteins

We propose that CED-6 might act redundantly with the AP2 complex to regulate internalization and correct localization of CED-1 through CME. The α subunit (*apa-2*) of AP2 interacts directly with CED-1 (Chen *et al*. 2013); however, which sequence it binds to in the cytoplasmic tail of CED-1 remains to be established. None of the other AP2 subunits or clathrin bind directly to CED-1 (Chen *et al*. 2013). However, we observed that the depletion of all 4 subunits affected CED-1 localization in a *ced-6* mutant background, with *dpy-23* (µ2) and *aps-2* (σ2) showing the strongest phenotype. Depletion of *epn-1* produced a mild CED-1 phenotype, suggesting that epsin is partly responsible for CED-1 localization, and may also bind CED-1 sorting signals. Epsin can bind to ubiquitinated endocytic cargoes through its ubiquitin-interacting motifs (Hawryluk *et al*. 2006; Kazazic *et al*. 2009). TRIM-21 poly-ubiquitinates CED-1; however, it remains to be established if CED-1 is ubiquitinated at the plasma membrane (Yuan *et al*. 2022). Epsin can also interact with AP2 and clathrin (Chen *et al*. 1998; Drake *et al*. 2000). As *epn-1* knockdown only produced a weak CED-1 phenotype, it is more likely that it affects CED-1 localization indirectly, through its interaction with AP2 and clathrin. DAB-1 is the sole homolog of ARH and Dab2, which are PTB domain proteins like CED-6. Interestingly, other PTB domain proteins play a role in recycling (Fu *et al*. 2012; Shah *et al*. 2013). However, depletion of *dab-1* does not result in CED-1 patches, which is consistent with the mislocalization of CED-1 being caused by an internalization/CME defect. Alternatively, DAB-1 does not bind the NPXY domain of CED-1.

Receptors that depend on several sorting signals for internalization include Yolkless, the LDLR, and LRP1. Specifically, Yolkless internalization depends on both CED-6, which recognizes an NPXY domain, and AP2, which binds to a dileucine sequence (Jha *et al*. 2012). Mutation of the dileucine sequence results in internalization of the Yolkless gene when CED-6 is present (Jha *et al*. 2012), and in the absence of AP2, oocytes still accumulate yolk (Parra-Peralbo and Culi 2011) indicating redundancy between CLASPs in mediating yolk uptake. LDLR contains two NPXY domains and is regulated by several adaptors, i.e. AP2, ARH, and Dab2 (Garcia *et al*. 2001; Keyel *et al*. 2006; Maurer and Cooper 2006; Eden *et al*. 2007; Ezratty *et al*. 2009). LRP1 has two NPXY domains, an YXXØ signal and two potential dileucine sequences (Li *et al*. 2001). In *C. elegans*, LRP1 internalization is mediated by 3 different sorting signals, through its NPXY domain, its single amino acid code HIC motif, and by an unknown motif recognized by EPN-1 (Kang *et al*. 2013).

Our study demonstrates a complex regulation of CED-1 localization and internalization through several redundant adaptor proteins. Furthermore, it stresses the need for CED-1 regulation even in the absence of engulfment of apoptotic cells. Further studies are needed to delineate the specific sorting signals in CED-1 recognized by the different adaptor proteins, and how the different adaptors affect each other to ensure correct localization and internalization of CED-1.

## Supplementary Material

jkae088_Supplementary_Data

## Data Availability

Strains are available upon request. The authors affirm that all data necessary for confirming the conclusions of the article are present within the article, figures, and tables. Supplemental material available at G3 online.
