# Supplementary material for: CED-6/GULP and components of the clathrin-mediated endocytosis machinery act redundantly to correctly display CED-1 on the cell membrane in Caenorhabditis elegans
Source: G3 (Bethesda). 2024 May 2;14(7):jkae088. doi: 10.1093/g3journal/jkae088 (PMC11228867; doi:10.1093/g3journal/jkae088)
Supplement: jkae088_Supplementary_Data [file jkae088_supplementary_data.pdf]

Supplementary material

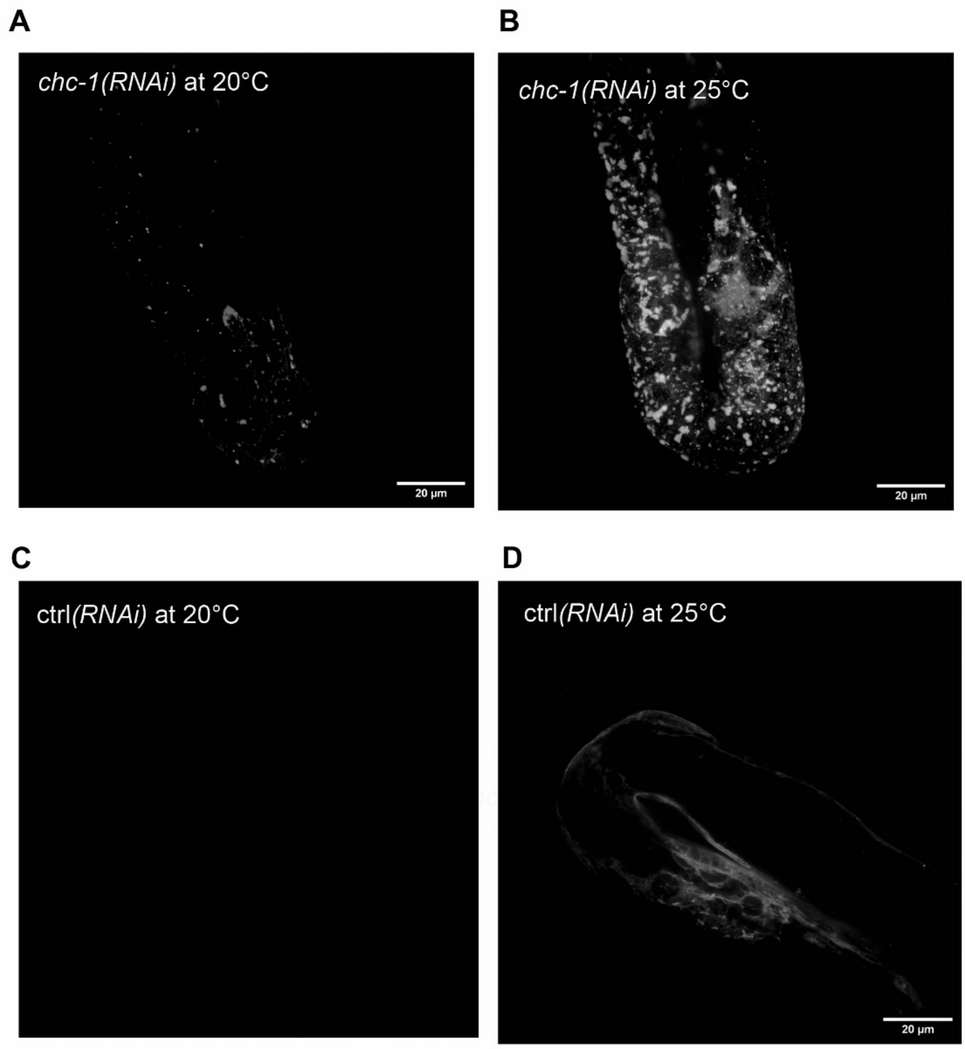

Figure S1. CED-1::GFP patches at 20°C and 25°C. A. CED-1::GFP patches seen following RNAi against *chc-1* at 20°C**. B.** CED-1::GFP patches seen following RNAi against *chc-1* at 20°C. **C**. CED-1::GFP in worms fed control RNAi at 20°C. **D**. CED-1::GFP in worms fed control RNAi at at 25°C. All pictures are maximum projections of z-stacks acquired using spinning disk confocal microscopy.

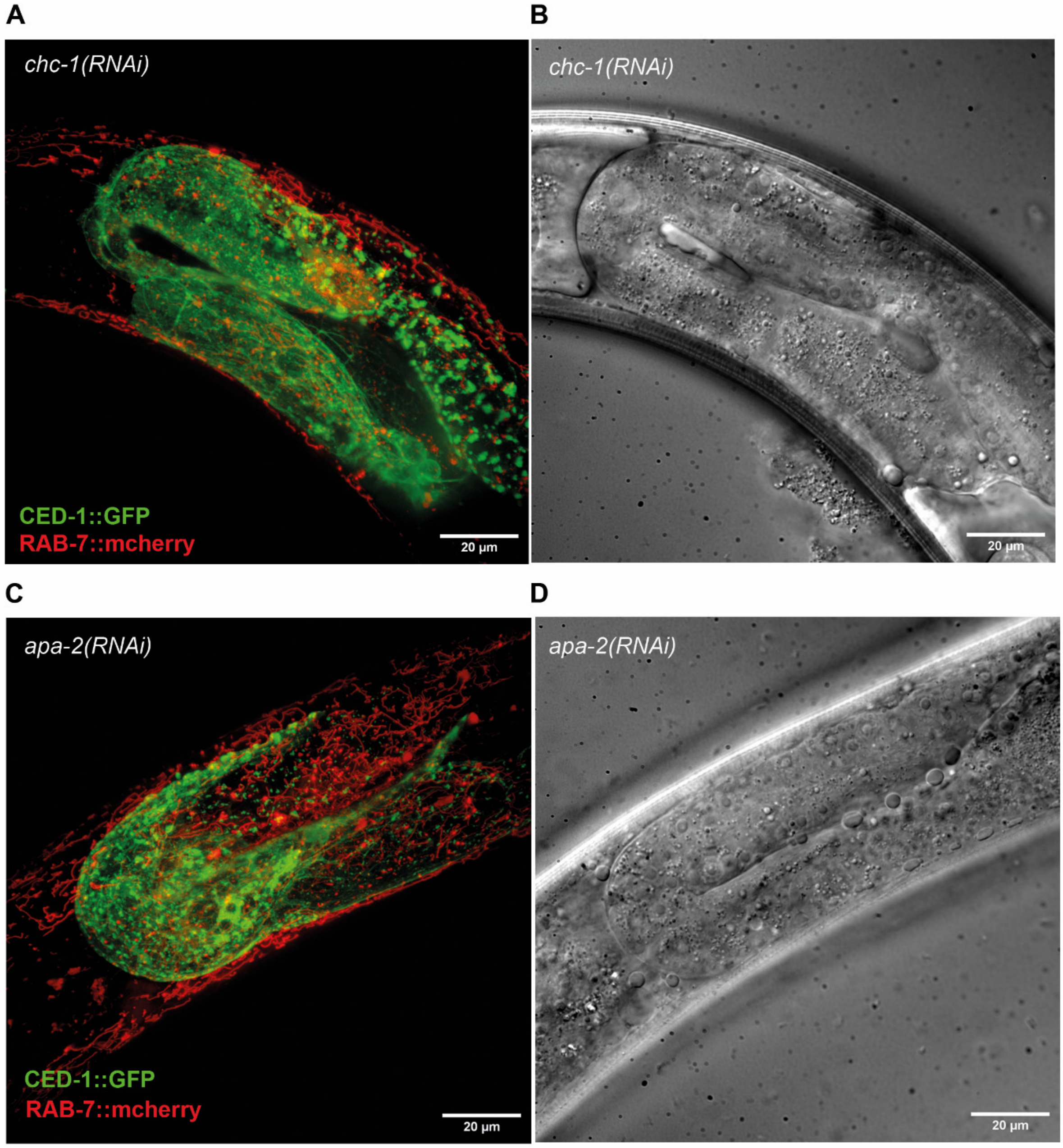

Figure S2. CED-1::GFP does not colocalize with the late endosome marker RAB-7. Maximum project of Z of confocal z-stacks. A. Image of CED-1::GFP puncta in *ced-6;ced-1::gfp* animals treated with RNAi against *chc-1.* CED-1::GFP puncta do not co-localize with RAB-7::mCherry positive late endosomes. **B.** Corresponding DIC image to A. **C**. Image of CED-1::GFP puncta in *ced-6;ced-1::gfp* animals treated with RNAi against apa-2. CED-1::GFP puncta do not co-localize with RAB-7::mCherry positive late endosomes. **D.** Corresponding DIC image to **C**.

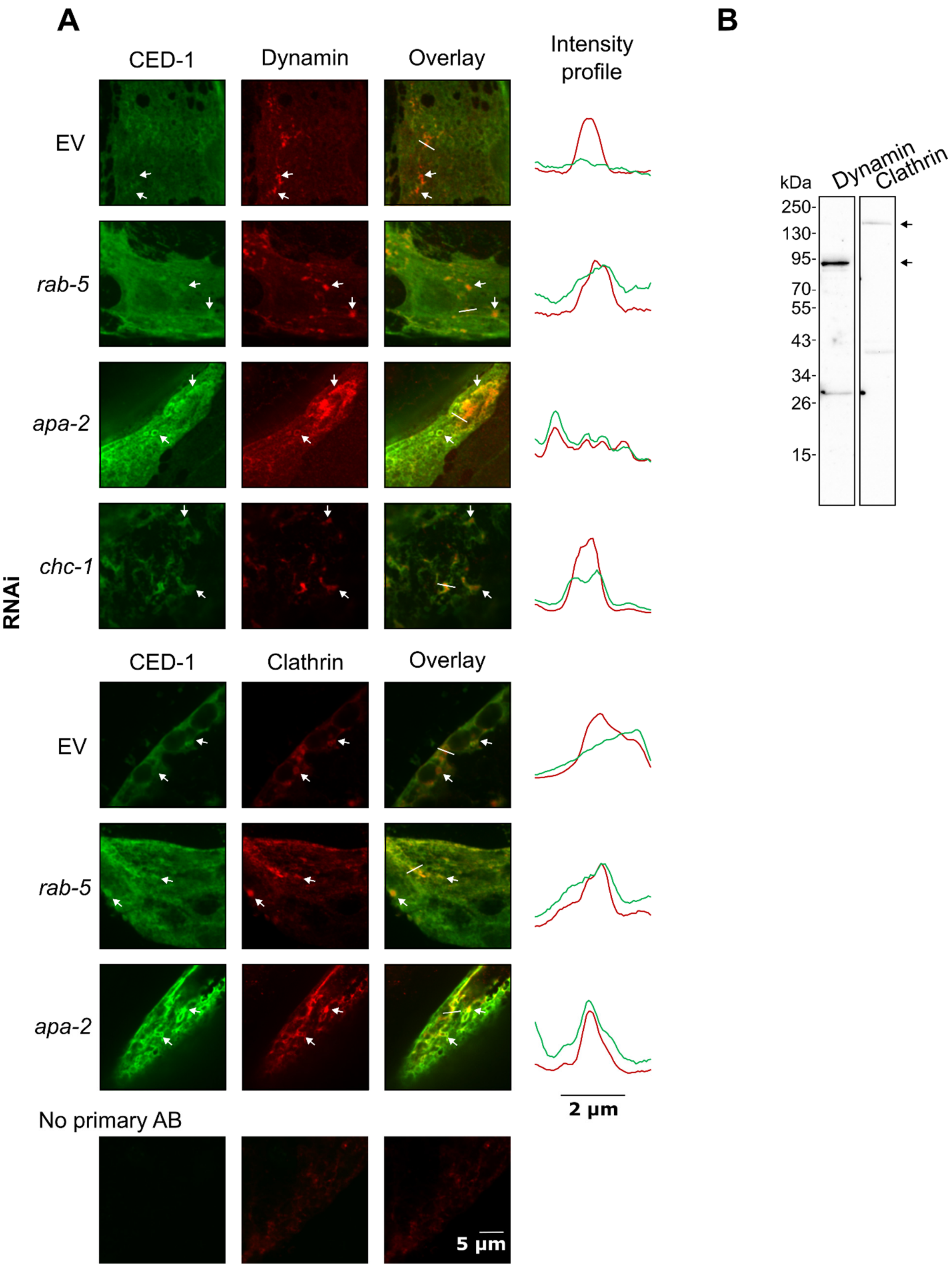
A
CED-1
Dynamin
Overlay
Intensity profile
EV
rab-5
apa-2
chc-1
RNAi
CED-1
Clathrin
Overlay
EV
rab-5
apa-2
2 µm
No primary AB
5 µm
B
kDa
Dynamin
Clathrin
250-
130-
95-
70-
55-
43-
34-
26-
15-

Figure S3. CED-1::GFP colocalizes with the early endosome markers DYN-1 and CHC-1. *C. elegans* worms were treated with RNAi against *rab-5*, *apa-2*, *chc-1*, or empty vector as controls. The worms were fixed, and stained with antibodies against dynamin or clathrin. Because the GFP signal from CED-1 is lost during the staining process, the GFP signal was amplified with an anti-GFP antibody. The worms were imaged using a spinning-disk confocal microscope with identical setting for all samples. Subsequently, the images were manually inspected to identify regions with sheath cells. **A.** Representative regions of CED-1 localization to early endosomes. All images are shown using identical brightness/contrast settings. Arrows indicate examples of early endosomes detected with anti-dynamin or anti-clathrin antibodies. Fluorescence signal intensity along indicated lines are shown to the right of each panel as examples of CED-1 levels in early endosomes. **B.** Western blot analysis of N2 *C. elegans* to confirm specificity of the two antibodies against dynamin (93.4 kDa) and clathrin (191.5 kDa).
